# Evaluation of the accuracy of deformable image registration on MRI with a physical phantom

**DOI:** 10.1002/acm2.12789

**Published:** 2019-12-06

**Authors:** Richard Y. Wu, Amy Y. Liu, Jinzhong Yang, Tyler D. Williamson, Paul G. Wisdom, Lawrence Bronk, Song Gao, David R. Grosshan, David C. Fuller, Gary B. Gunn, X. Ronald Zhu, Steven J. Frank

**Affiliations:** ^1^ Department of Radiation Physics The University of Texas MD Anderson Cancer Center Houston TX USA; ^2^ Department of Radiation Oncology The University of Texas MD Anderson Cancer Center Houston TX USA

**Keywords:** deformable image registration, deformable phantom, MR multimodality image registration

## Abstract

**Background and purpose:**

Magnetic resonance imaging (MRI) has gained popularity in radiation therapy simulation because it provides superior soft tissue contrast, which facilitates more accurate target delineation compared with computed tomography (CT) and does not expose the patient to ionizing radiation. However, image registration errors in commercial software have not been widely reported. Here we evaluated the accuracy of deformable image registration (DIR) by using a physical phantom for MRI.

**Methods and materials:**

We used the “Wuphantom” for end‐to‐end testing of DIR accuracy for MRI. This acrylic phantom is filled with water and includes several fillable inserts to simulate various tissue shapes and properties. Deformations and changes in anatomic locations are simulated by changing the rotations of the phantom and inserts. We used Varian Velocity DIR software (v4.0) and CT (head and neck protocol) and MR (T1‐ and T2‐weighted head protocol) images to test DIR accuracy between image modalities (MRI vs CT) and within the same image modality (MRI vs MRI) in 11 rotation deformation scenarios. Large inserts filled with Mobil DTE oil were used to simulate fatty tissue, and small inserts filled with agarose gel were used to simulate tissues slightly denser than water (e.g., prostate). Contours of all inserts were generated before DIR to provide a baseline for contour size and shape. DIR was done with the MR Correctable Deformable DIR method, and all deformed contours were compared with the original contours. The Dice similarity coefficient (DSC) and mean distance to agreement (MDA) were used to quantitatively validate DIR accuracy. We also used large and small regions of interest (ROIs) during between‐modality DIR tests to simulate validation of DIR accuracy for organs at risk (OARs) and propagation of individual clinical target volume (CTV) contours.

**Results:**

No significant differences in DIR accuracy were found for T1:T1 and T2:T2 comparisons (*P *> 0.05). DIR was less accurate for between‐modality comparisons than for same‐modality comparisons, and was less accurate for T1 vs CT than for T2 vs CT (*P* < 0.001). For between‐modality comparisons, use of a small ROI improved DIR accuracy for both T1 and T2 images.

**Conclusion:**

The simple design of the Wuphantom allows seamless testing of DIR; here we validated the accuracy of MRI DIR in end‐to‐end testing. T2 images had superior DIR accuracy compared with T1 images. Use of small ROIs improves DIR accuracy for target contour propagation.

## 1 INTRODUCTION

The use of magnetic resonance imaging (MRI) for radiation therapy simulations has gained popularity because it provides superior soft tissue contrast compared with conventional computed tomography (CT), potentially allowing more accurate target delineation without exposing the patient to unnecessary ionizing radiation. Co‐registration of two image sets using deformable image registration (DIR) before and during treatment is used to create a foundation for delineating targets^1^ to judge the need for adaptive radiation therapy. However, DIR errors in commercial software used with MRI have not been widely reported.

Use of image registration to improve the assessment of disease response is an area of active research.^2^ Notably, the magnitude of changes in tumor location and shape in actual patients over the course of treatment can be substantial or minimal,^3^, ^4^ with the accuracy of DIR varying accordingly. Moreover, registration errors made at an individual fraction of treatment affect the delivery of that fraction only, whereas systematic errors (including operator error) affect the delivery of all treatment fractions.^5^ Tyran et al.^6^ in evaluating the reliability of an MR‐guided online adaptive radiation therapy decision‐making process, concluded that daily review was not reliable for determining the need for adaptive radiation therapy, arguing that an online predicted plan, based on deformed and manually adjusted contours, should be generated for every fraction. Therefore, adequate DIR accuracy for propagating contours is essential to ensure the proper use of online adaptive radiation therapy.

Ger et al. evaluated a DIR system with synthetic images derived from patient longitudinal deformations and a porcine phantom with implanted markers.^7^ Tait et al. investigated the use of DIR in gynecologic brachytherapy to combine MRI guidance and CT‐based planning for optimizing placement of brachytherapy sources. In that study, DIR provided MRI guidance for CT‐based planning, which facilitated improved target volume delineation and dose escalation while minimizing toxicity to surrounding organs at risk.^8^ The stability of DIR for clinical purposes is affected by several factors, including image registration algorithms, input image quality, and regularization methods.^5^ The quality of image registration can be affected by other factors as well, including user experience and method.^9^ An MRI‐compatible phantom is needed that is sophisticated enough for “benchmarking” the uncertainties introduced by MRI‐only treatment planning and MR‐image‒guided radiation therapy.

Several MRI‐compatible phantoms have been developed, but to date all have had limitations. For example, Cunningham et al. developed a pelvis phantom to validate the MR‐image‐guided radiation therapy workflow.^10^ Unfortunately, this phantom cannot be used to test DIR accuracy because of the lack of quantitative measurements. De Brabandere et al. developed a CT‐ and MRI‐compatible prostate phantom that can be used to assess the accuracy of 3D image‐based reconstruction techniques.^11^ Niebuhr et al. developed the ADAM‐pelvis phantom, which is anthropomorphic, deformable, and multimodal.^12^ However, to date no standard physical phantom has been developed that allows end‐to‐end testing of the accuracy of DIR in MRI‐guided simulation and treatment delivery.

Regarding digital phantoms, the American Association of Physicists in Medicine (AAPM) task group report 132^5^ describes digital phantom images generated by ImSimQA software (Oncology System Limited, Shrewsbury, UK) that can be downloaded for MRI DIR testing. However, digital phantoms have many limitations, chief among them being that using digital phantoms would bypass the testing component of the MRI scanner, thereby making end‐to‐end testing of individual clinics with specific MRI units and DIR systems not possible.

The aim of this study was to use a previously reported physical phantom to evaluate the accuracy of DIR in T1‐weighted (T1) and T2‐weighted (T2) MRI and CT, with comparisons made within the same imaging modality (e.g., T1 vs T1 and T2 vs T2) and between different modalities (inter‐imaging modalities; e.g., T1 vs CT and T2 vs CT). We also assessed the accuracy of DIR between modalities by varying the sizes of regions of interest (ROIs) in the phantom volume. Our goal in this study was to demonstrate a method of benchmarking a DIR system to ensure the accuracy of contour propagation for adaptive radiation therapy.

## 2 METHODS AND MATERIALS

### 2.1 Phantom design

**Figure 1:**
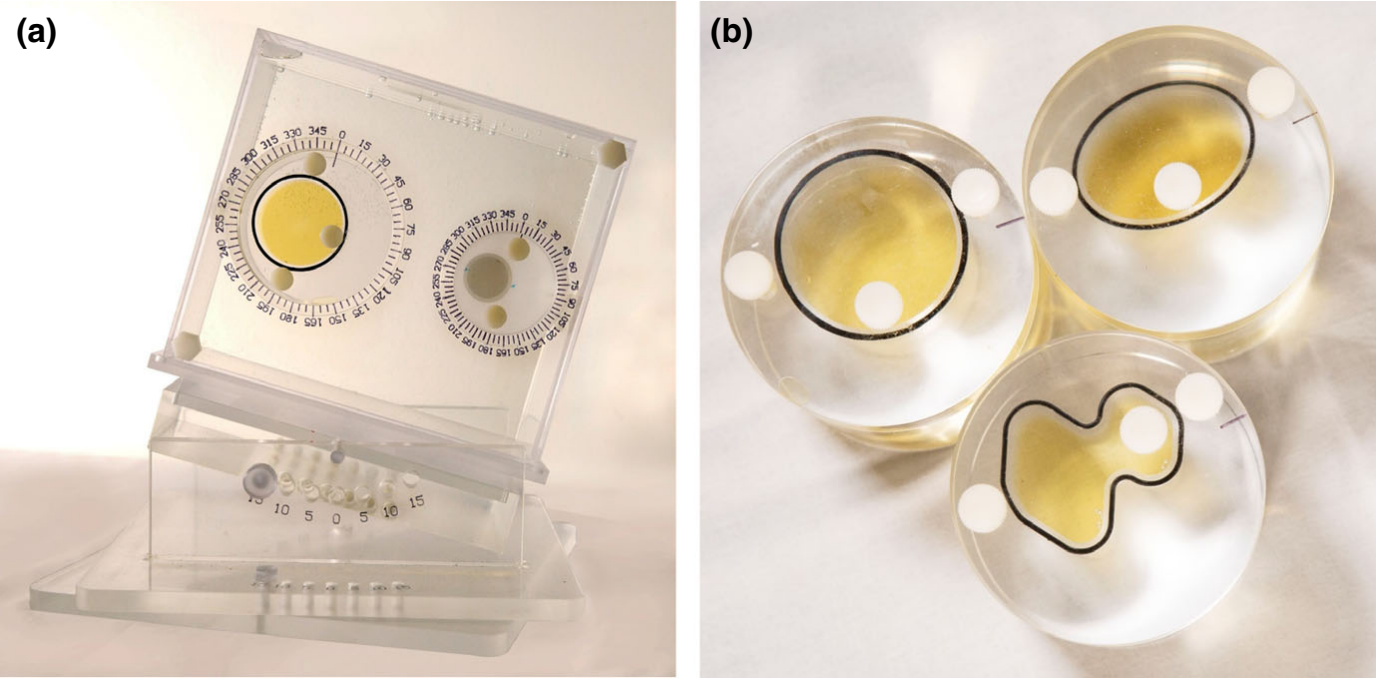
The Wuphantom consists of an acrylic frame and a base that can be tilted or rotated by 15°. For the tests reported here, the base is filled with water. The phantom has two cavities, a large one on the left and a small one on the right, and each cavity can be filled with inserts that can be filled with liquids to simulate different tissue densities. (a) The large insert (*left*) is filled with Mobil DTE oil, and the small insert (*right*) with a 4% agarose gel solution. (b) Three large inserts were made; the oval and irregular inserts represent contours deformed from the circle (baseline). White dots indicate caps through which liquids can be added to each insert.

The physical Wuphantom (US patent pending) was used previously to test the accuracy of DIR for CT and for CBCT.^13^, ^14^ This acrylic phantom includes a variety of inserts that simulate different tissue shapes and properties (Fig. 1). For MRI testing, the base of the Wuphantom was filled with water to make it visible on MRI. Deformations and changes in tumor locations are simulated by changing the rotations of both the phantom and its inserts. Three large cavity inserts were created in different shapes (circle, oval, and irregular) to simulate contours deformed from the original (baseline) shape (the circle). Both large and small circular inserts can be rotated to different degrees to mimic location changes in the X and Y directions for translation testing. For DIR testing, the inserts were rotated to simulate contour changes in the shape and location compared to the circle, which is usually used as the reference. Each large insert cavity was filled with Mobil DTE oil (ExxonMobil, Houston, TX, USA), the density of which (0.95 g/mL) represents that of fatty tissue, to volumes of 97.5 mL, 68.8 mL, and 61.1 mL. A smaller cavity on the right side of the phantom [Fig. 1(a)] has an insert containing 27.4 mL of 5% (w/v) agarose gel, to simulate tissues that are slightly denser than water. The image contrast was varied by using different agarose gel concentrations (0%, 0.5%, 1.0%, 2.0%, and 4.0%). The density of the 4.0% agarose gel is similar to that of prostate tissue (derived density of 1.036 g/mL), and its visualization characteristics on MRI are similar to those of prostate tissue.^15^ Although many other materials can be used to simulate human tissues under MRI,^16^, ^17^ we focused here on Mobil DTE oil and agarose gel to facilitate the study of low‐contrast subjects.

### 2.2 MR and CT image acquisition

MRI scans were obtained with a 1.5 T MRI Siemens MAGNETOM Aera scanner (Siemens, Inc., USA) with 8 × 2‐element flat head coils and a flat insert table. We selected 130 slices with an axial field of view of 25.6 cm and a superior–inferior slice direction (slice thickness of 2 mm) to cover the spatial region encompassing the entire phantom volume. CT images were also acquired with a Siemens Definition Edge CT scanner. Image acquisition parameters for CT and MRI are given in Table 1. All of the MR and CT images were transferred to a Velocity Workstation version 4.0 (Varian Medical Systems, Palo Alto, CA, USA).

**Table 1:** Acquisition parameters for MRI and CT images.

Acquisition technique	MRI T1	MRI T2	CT scan
Slice thickness (mm)	2.0	2.0	2.0
Sequence	Echo	Fast spin echo	
Flip angle	20	180	
TR (ms)	7.38	4800	
TE (ms)	4.77	80	
Pixel bandwidth (Hz)	400	300	
Acquisition pixel size (mm)	0.5	0.5	0.98
Matrix size	512 × 512	512 × 512	512 × 512
Slice spacing (mm)	0	0	0
Field of view (mm)	256	256	350
KV			120
mA			300

CT, computer tomography; KV, kilovoltage; TR, pulse sequence repetition time; TE, echo time; T1, T1‐weighted MRI sequence; T2, T2‐weighted MRI sequence.

**Figure 2:**
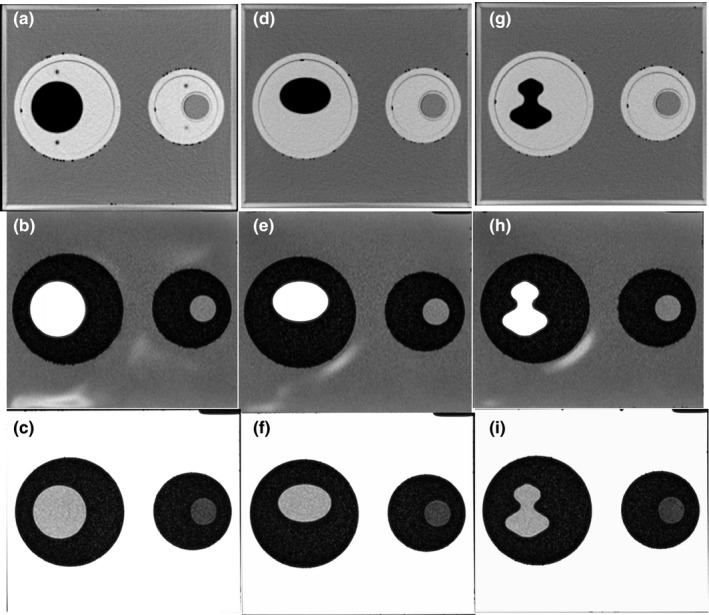
CT and MR images of the Wuphantom under similar viewing (window/level) conditions. Inserts (oval/irregular) at left are filled with DTE oil to simulate fatty tissue; inserts at right are filled with agarose gel (to simulate prostate tissue). Top row (a, d, g) is CT images; middle row (b, e, h) is T1 images; and bottom row (c, f, i) is T2 images.

To acquire reference images for both CT and MRI, the Wuphantom was placed on the base with 0° tilt and rotation, and the alignment marks (insert rotation, phantom tilting, and rotation) were set at 0°, the circular insert in the left large cavity was filled with DTE oil, and the smaller circular insert on the right cavity was filled with agarose gel. Images for DIR accuracy tests were acquired by replacing the circular insert with the oval or irregularly shaped inserts, and the inserts were rotated to different degrees to simulate location changes. We used 11 combined shape‐deformation scenarios to simulate both object deformation and location changes (Table 2). Sample images showed that the contrast inserts simulating the tumor and surrounding tissue in the Wuphantom were distinguishable on all CT and T1 and T2 MR images (Fig. 2).

**Table 2:** Eleven combined contour deformation scenarios to simulate both contour deformation and location changes.

Phantom insert rotation	Measured Inserts Volume, cm^3*^	
	Circle/Oval/Irregular	Agarose gel
Circle 0, agarose gel 0 (reference image)	100.1	48.1
Circle 90, agarose gel 45	100.5	48.1
Circle 180, agarose gel 180	105.4	46.2
Circle 270, agarose gel 225	98.1	47.3
Oval 0, agarose gel 0	68.3	46.8
Oval 90, agarose gel 0	68.9	47.4
Oval 180, agarose gel 0	68.9	46.9
Oval 270, agarose gel 0	68.1	47.4
Irregular 0, agarose gel 0	58.3	48.3
Irregular 90, agarose gel 0	58.8	48.1
Irregular 180, agarose gel 0	58.5	47.2
Irregular 270, agarose gel 0	58.7	46.8

All large inserts were filled with Mobil DTE oil (to simulate fatty tissue); smaller inserts were filled with agarose gel (to simulate prostate tissue).

Abbreviations: Circle, large insert with a circle shape; agarose gel, small insert with agarose gel; oval, large insert with an oval shape; irregular, large insert with an irregular shape.

### 2.3 Image registration

Image registration was done with Varian Velocity DIR software (version 4.0). Images were first registered using manual alignment by shifting and rotating the secondary image. Next, an ROI was drawn to encompass the whole phantom. Within this ROI, images were aligned first using Velocity rigid registration, which uses mutual information to align anatomy. DIR was done with the MR Correctable Deformable method. The MR correction applies a fade correction to the image to correct for shading artifacts resulting from heterogeneities in the magnetic field.

### 2.4 Contour propagation

**Figure 3:**
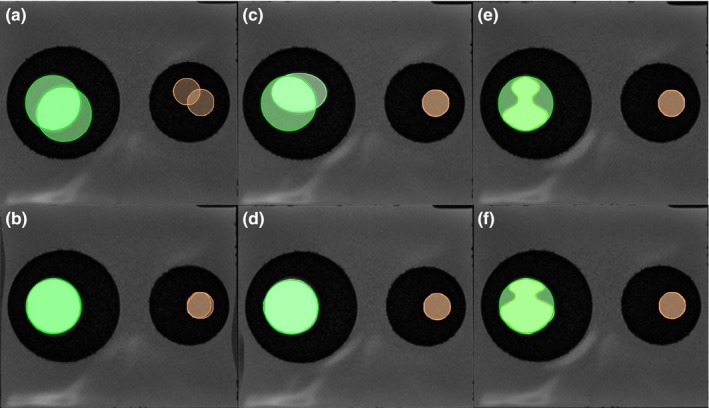
Contour shape and location changes before (upper) and after (lower) deformable image registration (DIR). (a, b) Circle, fatty tissue (left) and prostate tissue (right). (c, d) Oval, fatty tissue (left) and prostate tissue (right). (e, f) Irregular, fatty tissue (left) and prostate tissue (right).

Before DIR, contours were delineated for the large and small inserts [Fig. 3(a), (c), (e)] with a predefined threshold for both CT and MRI**.** This provides a “ground truth” for contours of various shapes for quantitative validation. After DIR, all of the contours were propagated from reference images (T1, T2, and CT) to images obtained from the other settings (secondary images). The overlay of the contours of various shapes after DIR is shown [Figs. 3(b), (d), and (f)].

### 2.5 DIR accuracy

Quantitative comparisons of the contours can be done with several metrics. Two commonly used approaches are the Dice similarity coefficient (DSC)^18^ and mean distance to agreement (MDA),^19^ also known as the mean distance to conformity. The DSC is defined as the ratio of twice the overlap of two structures (A and B) over the sum of their volumes and is widely used in DIR comparisons. DSC (A, B) = 2(A ∩ B)/(A + B). The DSC ranges from 0 to 1, with higher values indicating matter match or agreement between two structures. MDA is a geometric parameter that measures the mean voxel shortest distance from the surface of one structure to another (ideal = 0 mm).

### 2.6 Statistical analysis

All DSC and MDA data were compared between the MRI and CT scans in paired sample analyses; Wilcoxon matched‐pair nonparametric tests^20^ were used to evaluate differences between MRI and CT registration. A probability value of *P* ≤ 0.05 was considered statistically significant. All statistical analyses were calculated using R statistical software (R Foundation for Statistical Computing, Vienna, Austria).

## 3 RESULTS

**Figure 4:**
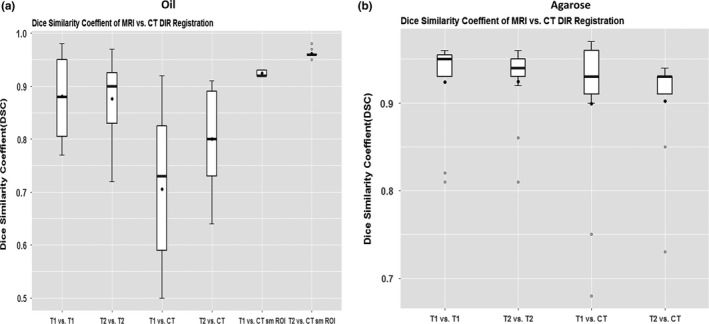
Dice similarity coefficients for MRI vs CT deformable image registration for (a) Mobil DTE oil (simulating fatty tissue) and (b) agarose gel (simulating prostate tissue).

**Figure 5:**
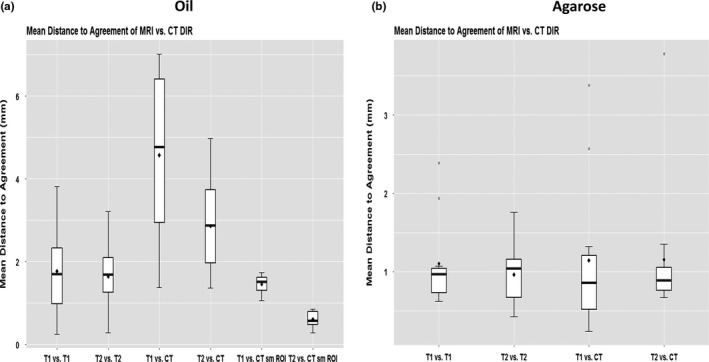
Mean Distance to Agreement (MDA), in mm, for (a) Mobil DTE oil simulating fatty tissue and (b) agarose gel simulating prostate tissue.

Registration of images obtained with the same modality (CT vs CT, T1 vs T1, or T2 vs T2) showed no differences in DIR accuracy for the T1:T1 and T2:T2 comparisons (*P *> 0.05). For both the T1:T1 and T2:T2 comparisons, mean (±SD) DSC values for fatty tissue (oil) were 0.88 ± 0.08, and those for prostate (agarose gel) were 0.92 ± 0.05 (Table 3). Comparisons in DSC values for MRI vs CT DIR are also shown in Table 3 and illustrated graphically in Fig. 4. MDA values differed slightly in the T1:T1 and T2:T2 comparisons for both fatty tissue and prostate (Table 3). The mean DSCs for between‐modality DIR were similar for T1 vs CT and for T2 vs CT for fatty tissue (T1 vs. CT = 0.71, T2 vs. CT = 0.80) and for prostate (both T1 vs CT and T2 vs CT = 0.90) (Table 3). As for the MDA values, the mean MDA for prostate was similar in T1 vs CT and T2 vs CT comparisons (1.15 mm and 1.16 mm), but the mean MDA values for fatty tissue were different for T1 vs CT (4.57 mm) and T2 vs CT (2.86 mm) (Table 3, Fig. 5). In other words, DIR accuracy was lower for between‐modality comparisons (T1 or T2 vs CT) than for same‐modality comparisons (T1 vs T1 or T2 vs T2), and the accuracy was also lower for T1 sequences than for T2 sequences (*P* < 0.001) for both fatty and prostate tissues.

**Table 3:** Measures of accuracy of deformable image registration within and between imaging modalities.

Modality	DSC_fatty tissue_	MDA_fatty tissue_	DSC_prostate_	MDA_prostate_
T1 vs T1	0.88 ± 0.08	1.77 mm ± 1.1 mm	0.92 ± 0.05	1.1 mm ± 0.53 mm
T2 vs T2	0.88 ± 0.08	1.64 mm ± 0.8 mm	0.92 ± 0.04	0.96 mm ± 0.37 mm
T1 vs CT	0.71 ± 0.14	4.57 mm ± 1.95 mm	0.90 ± 0.09	1.15 mm ± 0.92 mm
T2 vs CT	0.80 ± 0.09	2.86 mm ± 1.08 mm	0.90 ± 0.06	1.16 mm ± 0.85 mm

T1, T1‐weighted MRI sequence; T2, T2‐weighted MRI sequence; CT, computer tomography; DSC, Dice similarity coefficient; MDA, mean distance to agreement.

**Figure 6:**
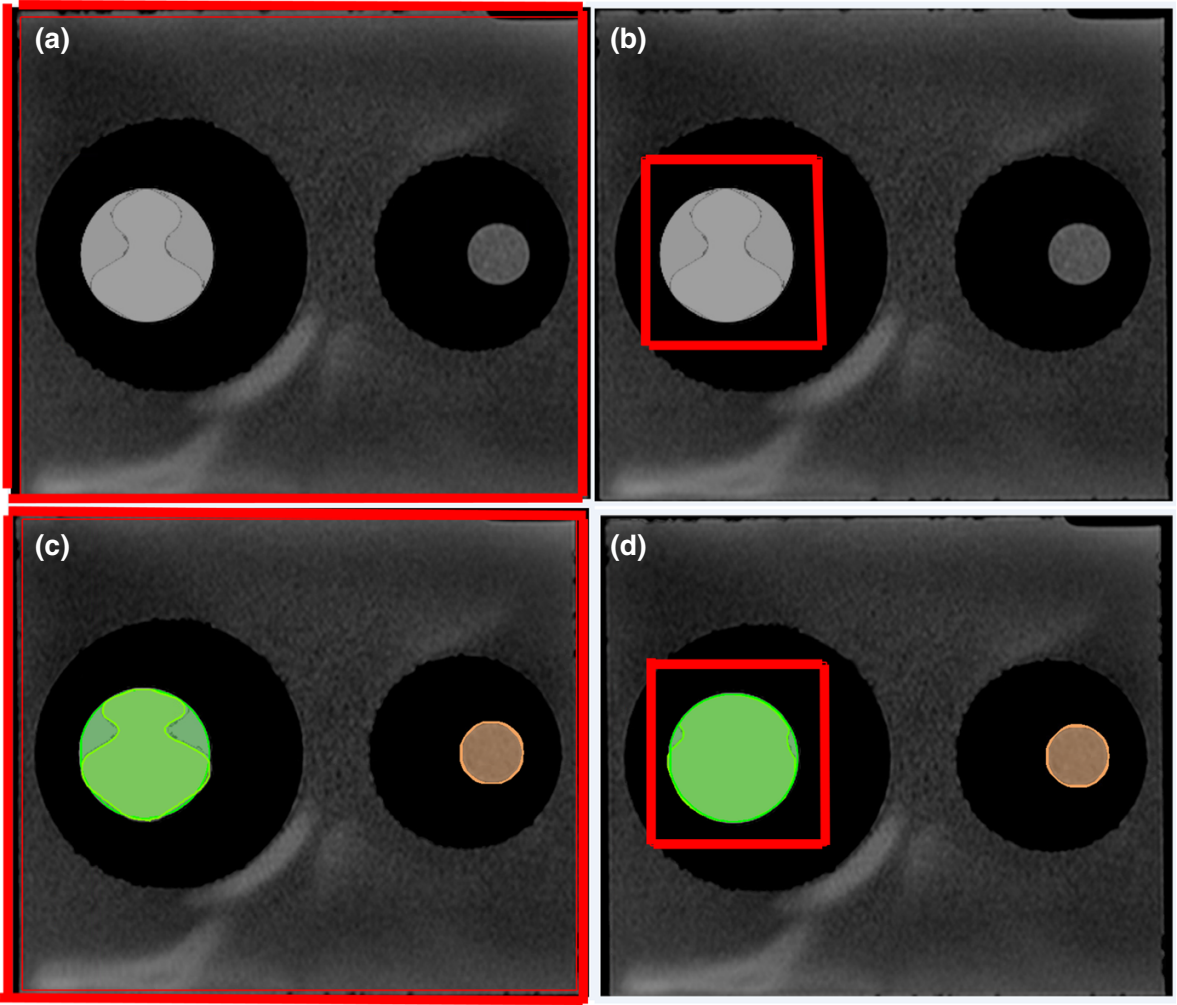
The large red boxes (a, c) represent large regions of interest (ROIs), and the smaller red boxes (b, d) represent smaller ROIs. (c, d) Overlays of deformed contours in the large ROI (c) vs the smaller ROI (d). In all cases, the insert on the left is filled with Mobil DTE oil to simulate fatty tissue.

We also compared the effects of ROI size (large vs small) on between‐modality DIR (Fig. 6). The volume of the large ROI, which encompassed the entire phantom, was 20 cm × 20 cm ×14 cm; the volume of the small ROI was 8 cm × 8 cm ×7 cm. The smaller ROI was drawn around the oil‐filled (fatty tissue) insert to simulate a CTV. Using a small ROI improved the DIR accuracy for both T1 and T2 images. For T1 images: DSC_large_ = 0.71, DSC_small_ = 0.92 [*P* < 0.0003]; and MDA_large_ = 4.5 mm, MDA_small_ = 1.5 mm [*P* < 0.0003] for T2 images: DSC_large_ = 0.8, DSC_small_ = 0.96 [*P* < 0.001]; and MDA_large_ = 2.86 mm, MDA_small_ = 0.61 mm [*P* < 0.0001].

## 4 DISCUSSION

We report here use of the Wuphantom to quantitatively evaluate the accuracy of DIR for CT and two sequences of MRI. The tests included both within‐modality comparisons (T1 vs T1 and T2 vs T2) and between‐modality comparisons (T1 vs CT and T2 vs CT). DIR was less accurate for between‐modality than for same‐modality comparisons. All of the results (except for T1 vs CT) were within the AAPM's recommended thresholds (DSC > 0.8 and MDA < 3 mm). DIR accuracy was better for T2 images than for T1 images on between‐modality comparisons. We also found that using a small ROI improves the accuracy of DIR for target contour propagation.

The DIR process has uncertainties regardless of the algorithm chosen. For areas with very low tissue contrast, registration can be prone to inaccuracies.^21^ Registration can be defined by nine criteria:^22^ dimensionality, nature of the registration basis, nature of the transformation, domain of transformation, interaction, optimization procedure, modalities involved, subject, and object. Therefore, registration tests using a physical phantom are essential to ensure the accuracy of the entire imaging workflow. In our study, the Wuphantom was used to evaluate DIR of three shapes: circular, oval, and irregular. Nevertheless, phantom DIR testing is at best a representation of the accuracy of a DIR system. The actual accuracy of DIR for treatment simulation and delivery with patients may vary in different ways.

Buch et al. evaluated the influence of MRI scanning parameters on texture analysis features and found that variations in MRI acquisition led to significant differences in many texture features.^23^ Moreover, the tumor texture and image intensities were different before vs after treatment. The histologic type and anatomic location of tumors (e.g., head and neck, brain, or lungs) can also affect DIR accuracy. We recommend that DIR accuracy be further evaluated as a function of tumor contrast in images acquired using different protocols and different materials that simulate a broader range of tissue characteristics.

MR‐linac systems, which combine MRI with a linear accelerator, can provide images of patient anatomy in real time. Although rigid and non‐rigid deformations are available before treatment, a full intensity‐modulated radiation therapy optimization system is needed that can adapt to ongoing updates on anatomic data during fraction delivery.^24^ Registration for treatment delivery and online treatment adaptation requires accurate DIR to ensure accurate treatment. The Wuphantom may be useful for validating DIR accuracy on the MR‐linac as well.

Our study did have some limitations. We used a standard head MRI T1 and T2 scanning protocol for the reference and secondary images. We also did not fully evaluate the registration results when the scanning protocol changed (e.g., proton density, diffusion‐weighted images, or slice thickness changes). Moreover, a physical phantom usually has rather simple geometry and correspondingly simple deformations. Even though the DIR in the current study was found to be quite accurate for the evaluated scenarios, we acknowledge that uncertainty still exists in the DIR process for patient‐specific images. Indeed, the phantom is more useful for detecting systemic error of a DIR system than for evaluating the accuracy of a clinical case.

## 5 CONCLUSIONS

We quantitatively evaluated the accuracy of DIR for MRI and CT. For between‐modality comparisons (T1 vs CT or T2 vs CT), T2 imaging performance was better than T1 imaging performance. Use of a smaller ROI was found to improve the accuracy of DIR for target contour propagation. The AAPM recommends that a physical phantom be used for end‐to‐end testing to account for variations in the imaging chain; we believe that our work with the Wuphantom is an important contribution to such testing.

## CONFLICTS OF INTEREST

A patent related to wuphantom has been filed.
